# A systematic review of qualitative studies examining barriers and facilitators to orthopaedic surgeon engagement with patient-reported outcome measures data

**DOI:** 10.1186/s41687-024-00820-x

**Published:** 2024-12-18

**Authors:** Emma L. Heath, Ian A. Harris, Lorena Romero, Ilana N. Ackerman

**Affiliations:** 1 School of Public Health and Preventive Medicine, Monash University, 553 St Kilda Road, Melbourne, VIC 3004 Australia; 2 South Australian Health and Medical Research Institute, North Terrace, Adelaide, South Australia 5000 Australia; 3 School of Clinical Medicine, UNSW Medicine and Health, UNSW Sydney, Sydney, New South Wales 2052 Australia; 4 Whitlam Orthopaedic Research Centre, Ingham Institute for Applied Medical Research, 1 Campbell Street, Liverpool, New South Wales 2170 Australia; 5 The Alfred Hospital, 55 Commercial Rd, Melbourne, VIC 3004 Australia

**Keywords:** Patient-reported outcomes, Orthopaedic surgeon engagement, Systematic review

## Abstract

**Background:**

Orthopaedic surgeon engagement with patient-reported outcome measures (PROMs) data has not been comprehensively evaluated, despite increasing uptake of orthopaedic PROMs programmes globally. The aim of this review was to systematically identify, appraise and synthesise qualitative evidence on barriers and facilitators to orthopaedic surgeons’ engagement with PROMs data and their use of these data to support clinical practice.

**Methods:**

Six databases (MEDLINE, EMBASE, COCHRANE CENTRAL, PSYCINFO, CINAHL and EMCARE) were searched from January 2000—March 2024 to identify potentially eligible qualitative studies. Established systematic review methods were used for screening and data extraction, applying PRISMA guidelines. Quality assessment was undertaken using the Joanna Briggs Institute tool for qualitative research.

**Results:**

Eight studies were eligible for inclusion; of these, five studies were qualitative and three studies were mixed-method designs incorporating a qualitative component. Three studies were specific to orthopaedic surgeons and the remaining five studies comprised of mixed samples of health professionals including orthopaedic surgeons. Only one study was classified as being of high methodological quality. Key barrier themes for orthopaedic surgeons were logistical issues, difficulty interpreting and understanding PROMs, and scepticism of the value of PROMs in clinical care. Key enabler themes included improvements to PROMs infrastructure, surgeon education around the potential value, uses and interpretation of PROMs data, aggregate reporting of PROMs data and early involvement of surgeons in the planning and development of PROM systems.

**Conclusion:**

While these studies highlight some practical considerations and opportunities that can be addressed through clinician education, there is little high-quality evidence on factors that influence orthopaedic surgeon engagement with PROMs data. Robust qualitative research is needed to better inform tailored support and assist surgeons in integrating PROMs data within orthopaedic care.

**Supplementary Information:**

The online version contains supplementary material available at 10.1186/s41687-024-00820-x.

## Background

The collection of patient-reported outcome measures (PROMs) data is increasingly common within orthopaedic care settings [1, 2]. PROMs are standardised, validated questionnaires completed by patients to ascertain perceptions of their pain, health status, disability, and health-related quality of life [3, 4]. With growing momentum towards the routine collection of PROMs data by clinicians and arthroplasty registries, there is a clear opportunity for orthopaedic surgeons to use these data to inform their clinical practice. Available literature indicates that there is potential for surgeons to use PROMs data for benchmarking of patient outcomes and as a performance appraisal tool [5, 6]. Specifically, PROMs data can be used by surgeons for setting thresholds for surgery, for identifying suboptimal post-operative recovery, and as indicators of overall surgical quality [7–9]. At the patient level, PROMs data can also facilitate a patient-centred approach to clinical care by allowing for shared decision making [10]. More recently, PROMs data have been used at the surgeon level to provide key information on component and prothesis comparisons [11–13]. Taken together, PROMs data offer surgeons a valuable opportunity for informing and improving their clinical practice [7–9].

For PROMs data to be of most value, a better understanding of the factors that assist (and impede) orthopaedic surgeons to use PROMs data within clinical care is needed. Boyce et al. conducted a systematic review which examined health professionals’ views of PROMs data within healthcare [14]. This review determined that PROMs were generally perceived to be useful for health professionals’ decision making; however, barriers such as technology and difficulties in interpretation of PROMs data were common. The authors concluded that improvement in the uptake of PROMs data may be facilitated by engaging professionals early in setting up data collection processes and improving timely access to PROMs data by optimising technology. Further profession-specific research was also recommended, to better understand the motivations of different health professional groups. To our knowledge, there has been no review of research regarding PROMs engagement that is specific to orthopaedic surgeons or conducted within orthopaedic settings. The aim of this systematic review was to identify, appraise and synthesise available qualitative evidence on the barriers and facilitators to orthopaedic surgeons’ engagement with PROMS data and their use of PROMs data to support clinical decision making and patient care.

## Methods

### Study design

A systematic literature review was undertaken. The protocol was registered on the PROSPERO International Prospective Register of Systematic Reviews (registration number CRD42023412776). The review is reported according to the Preferred Items for Systematic Reviews and Meta-Analysis (PRISMA) 2020 statement.

### Search strategy

Electronic literature searches were undertaken in six key databases (MEDLINE, EMBASE, COCHRANE CENTRAL, PSYCINFO, CINAHL and EMCARE). With specialist research librarian assistance and orthopaedic surgeon input, a comprehensive search strategy was designed and developed for each database (Table S1, Supplementary File). The search strategies were limited to papers published in English from January 2000 to May 2023, which spans the period of major growth in PROMs research. All database searches were re-run in March 2024, to identity any further published studies. The reference lists of previously identified key literature and systematic reviews identified in the initial search yield were hand searched to identify any additional primary studies.

### Eligibility criteria

Eligible studies were primary qualitative or mixed-methods designs that reported on perceived barriers and enablers to the use of PROMs and/or engagement with PROMs data by orthopaedic surgeons. We included studies involving either mixed samples of health professional participants that included orthopaedic surgeons or samples of orthopaedic surgeon participants only. There were no specific exclusions based on area of clinical practice and studies from any geographical location and any clinical setting were eligible. Review papers and studies that were published only as conference abstracts were not eligible for inclusion.

Two reviewers (ELH, INA) independently screened the titles and abstracts of all identified studies using Covidence software (Veritas Health Innovation Ltd, Melbourne, Australia) to determine potential eligibility for inclusion. The full-texts of all potentially eligible studies were then reviewed independently by each reviewer to confirm eligibility. The reference lists of all included studies were also checked for potentially relevant studies. At each review stage, any discordance regarding eligibility was discussed to reach consensus, where required.

### Data extraction

Two reviewers (ELH, INA) independently extracted data from each included study using a customised template. The following data were extracted: study characteristics (year of publication, country, study design, study setting, sample size), methodology (type of qualitative design and approaches used for data collection and analysis) and clinician characteristics (profession, years of clinical experience, gender). For studies that involved only orthopaedic surgeon participants, key themes, subthemes and supporting quotes were extracted. Themes, subthemes and supporting quotes were also extracted from studies that involved mixed samples of health professionals only where it was clear that these were derived only from orthopaedic surgeons. These themes were reported separately to those derived from the studies specific to orthopaedic surgeons, to avoid potential bias from the inclusion of other health professional participants when the themes in the mixed sample studies. We also extracted supporting quotes attributed to ‘trauma surgeons’ to avoid missing data pertaining to orthopaedic surgeons (in some countries, both terms are used to describe surgeons who perform bone and joint surgery). Data extracted by each reviewer were compared to identify any inconsistencies, which were resolved through discussion where required.

### Quality assessment of included studies

The methodological quality of all included studies was assessed independently by two reviewers (ELH, INA) using a validated critical appraisal tool for qualitative research from the Joanna Briggs Institute [15]. The assessment tool included 10 items that assessed the research methodology against the philosophical perspective, research questions, methods of collection and results. It also considered factors such as ethics and representation of participants’ voices within the included studies. Any discrepancies in risk of bias assessment were resolved through consensus to achieve an agreed rating for each included study.

### Data synthesis

Study and participant characteristics for each of the included studies were reported descriptively. Key themes and subthemes relating to orthopaedic surgeons were classified into barriers and enablers by using all reported barriers and enablers data from the papers; the themes were then used to consider opportunities and potential actions.

## Results

### Search yield and included studies

**Fig. 1:**
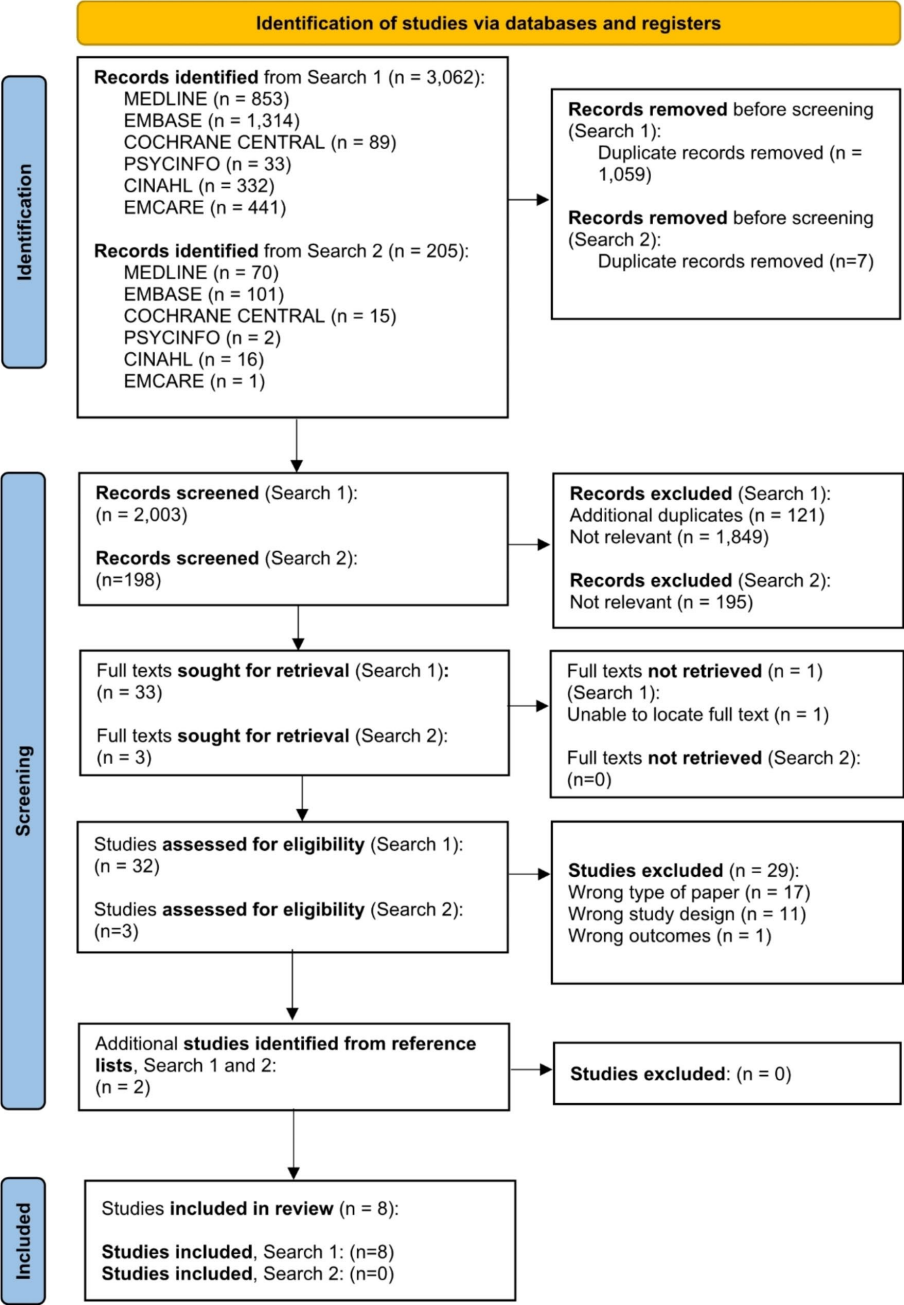
PRISMA 2020 flow diagram

The study selection process is summarised in Fig. 1. The process of removing duplicates and screening titles, abstracts and full texts yielded eight studies that were eligible for inclusion. The characteristics of the included studies are summarised in Table 1. The included studies were from the United States of America (*n* = 6), Finland (*n* = 1) and Ireland (*n* = 1) and were published between 2014 and 2023. Of the included studies, five studies were solely qualitative in design, two were qualitative studies nested within larger randomised controlled trials and one study incorporated a qualitative component within a larger feasibility and usability testing study of an electronic PROMs information system. The number of study participants ranged from nine to 30. Five studies involved mixed samples of health professionals and three studies involved orthopaedic surgeons only; the latter involved samples of two to 11 orthopaedic surgeon participants. Orthopaedic surgeon participants had a range of clinical experience (from less than five years to over 19 years).

**Table 1:** Characteristics of the included studies

Study	Country	Study design	Setting	Participants	Data collection approach	Data analysis approach	Years of clinical experience; Gender
Boyce (2014)	Ireland	Qualitative research study nested within a larger RCT	Hospital	11 high-volume hip replacement surgeons	Face-to-face interviews	Framework approach	Years of clinical experience not specified; 11 male participants (100%)
Driscoll (2022)	USA	Qualitative study (not further specified)	Academic medical centre	9 surgeons from 8 specialties including trauma	Semi-structured interviews	Thematic analysis	Years of clinical experience not specified; gender not specified
Jansson et al. (2019) [28]	Finland	Qualitative exploratory study	Joint replacement centre in a tertiary-level university teaching hospital	4 surgeons, 2 anaesthesiologists, 10 nurses, 4 physiotherapists	Semi-structured interviews	Inductive content analysis	Years of clinical experience not specified; ‘majority of participants were female’ (not further specified)
Lavallee (2023)	USA	Qualitative research nested within a pragmatic cluster RCT	Medical school-based practices, community-based arthroplasty specialty practices, community-based general orthopaedic practices	9 orthopaedic surgeons	Semi-structured interviews	Conventional content analysis	Years of clinical experience: <5 years: 3; 5–10 years: 3; 11–19 years: 0; >19 years: 3; gender not specified
Mou (2022)	USA	Exploratory qualitative study	3 academic medical centres	30 surgeons from 5 subspecialities including orthopaedic surgery	Semi-structured interviews	Thematic analysis	Years of clinical experience: mean 14 for bariatric, mean 11 for breast surgeons, mean 12 for rhinologists, mean 12 for orthopaedic surgeons, mean 8.8 for plastic surgeons; 83% male for bariatric, 14% male for breast surgeons, 40% male for rhinologists, 100% male for orthopaedic surgeons, 40% male for plastic surgeons
Rothrock et al. (2019) [29]	USA	Alpha and beta testing with semi-structured interviews	3 orthopaedic trauma clinics in academic medical centres	11 clinic staff including 5 surgeons and physicians assistants (also specified on page 9 that 5 clinicians across 4 sites completed the interview)	Semi-structured interviews	Not specified	Not specified
Whitebird (2022)	USA	Cross-sectional qualitative study	Single health system in an urban setting	9 orthopaedic surgeons and 2 orthopaedic leaders	Semi-structured interviews	Conventional content analysis	Years of clinical experience: median 13 years; 8/9 surgeons were men, ½ orthopaedic leaders were mean
Zhang (2019)	USA	Qualitative study (not further specified)	Orthopaedic and oncology clinics	2 orthopaedic surgeons and 9 oncology health care providers	In-depth interviews	Thematic analysis	Years of clinical experience not specified; 4 male participants (36%) and 7 female participants (64%)

### Quality assessment

Only one included study [16] was assessed as being of high methodological quality, meeting all quality assessment checklist criteria (Table S2, Supplementary File). Two other studies met all checklist criteria, apart from one item (congruity between the stated philosophical perspective and the research methodology). The remaining five studies were of variable quality.

### Reported barrier and enabler themes

The key themes emerging from the orthopaedic surgeon studies are summarised in Table 2. A summary of the barrier and enabler themes from all included studies (*n* = 8), mapped to opportunities and potential actions, is detailed in Table 3. The barrier and enabler themes are described below, first detailing the orthopaedic surgeon specific studies (*n* = 3) and then the mixed sample studies (*n* = 5).

**Table 2:** Views of orthopaedic surgeons: key themes emerging from each study

Study	Themes	Subthemes	Supporting quotes
Boyce (2014)	Conceptual—understanding PROMs	Subjective measurement	‘Getting patients to fill out forms is grossly inaccurate in my book…the patient 9 times out of 10 wouldn’t understand what hip pain is’ (S9) ‘There is some subjective element but it is a reasonably validated objective assessment’ (S2). ‘Well they are partly objectified, aren’t they?’ (S11) ‘I suppose the difference maybe with my results is the difference between the maybe more objective measures and the subjective measures’ (S5)
		PROMs versus satisfaction	‘Patient satisfaction in a sense is a balance between what their expectations were beforehand and what they achieved afterwards’ (S10) ‘You know there is one outcome there on how much the patient likes the outcome as I like to call it’ (S2) ‘When they are not perfect, they manifest that by saying they are quite poor’ (S7)
		PROMs versus clinical data	Clinically I see very very very few problems and very few dissatisfied patients…that is just wrong. I am sorry I just can’t accept that’ (S10)
	Methodological—measurement decisions, measurement accuracy and interpretation	Focus and variability	‘You should concentrate on operations that have dubious results’ (S8) ‘The increments between each surgeon are tiny…I mean your spread there between top and bottom is only six points’ (S7)
		Timing	‘To see if there was any differences at four to six weeks’ (S4) ‘The other thing is the timing is critical because one would generally not measure anything in hip surgery and knee surgery for at least one year’ (S11)
		Choice of measures	‘That score has issues with validity for certain age groups’ (S1) ‘The patient might perceive it as a complication but it is not, it is part of the normal process’ (S8) ‘You know it has to be patients with a problem after surgery that is directly related with the surgery’ (S10)
		Interpretation	‘Unless I was able to compare myself against somebody else who does things quite differently’ (S2) ‘I mean strictly speaking someone that is at the tail end should be at the tail end in all three’ (S7)
		Validity	‘Something is wrong somewhere: either they have problems and they are not telling me or else there is something odd in data collection’ (S10) ‘Even if you adjust them it is not going to give you the proper information’ (S1)
	Practical issues with collecting and using the data	Time	‘If I had time, maybe. I don’t have time. I mean, I have continuous ideas…and am…let’s say resolutions to measure outcomes better and more often and all the rest of it but we don’t have the time like and we don’t have the staff’ (S11)
		Support	‘No interest. No support. No help. No funding’ (S2) ‘We don’t have anything strictly audit related because the big problem with the hospital audits is the information gathering is poor’ (S7) ‘You need generally a political will to get it because it can achieve nothing but to cost them more’ (S2) ‘You need software, you need somebody to analyse it’ (S3) ‘…that takes help, statistical help’ (S4)
	Attitudinal	Value	‘There have been a lot of high profile problems in recent times and maybe these kind of problems would have been spotted sooner if we were collecting this type of data’ (S5) ‘You see your patients and they are happy but in general terms you don’t know how you are performing compared to your peers’ (S4)
		Undecided	‘That is kind of a relatively disappointing figure, I would have thought and not just mine, I think the overall is kind of a little bit disappointing. Why it is? I am not sure’ (S3)
		No value	‘I just think there is a lot of effort being put in there for not a lot of surgical gain from my perspective’ (S8)
	Impact	Impact	‘I am going to try and do it better’ (S4) ‘I went off for a few days and started thinking about things so even though my results would appear not to be brilliant, it was very beneficial for me’ (S7)
		No impact	‘I seem to be in the middle there and I wouldn’t be changing what I do on the basis of it’(S2) ‘Unfortunately, it does not provide me with one iota that helps me make my next score any better’ (S10)
Lavallee (2023)	Acceptability	N/A	“I think this is very, very important data, and it’s really wonderful to have visually. I think people are really helped by it.” E “Giving some objective numbers to the patient [sic] symptoms, I think, is very, very important.” I “And some patients are like, well, I already knew I wanted surgery anyways, so this is not changing my mind or making an difference in terms of my decision or outcomes.” D
	Patient characteristics	N/A	“I use the report most commonly on…patients that need a little bit more information, they’re kind of skeptical to begin with.” C
	Communication goals	Education	“At that first visit, using the pain numbers and the function numbers to explain where they are in their disease process in relation to other patients with the same disease.” I “I really go over what their predicted score changes will be and show them what comorbidities are affecting them that we can’t change. So, like low back pain, or whatever.” A
		Expectation setting	“We’re setting expectations that they will improve. But trying to make those expectations for improvement realistic.” B “You’d have a lot of pain relief. You’d have much improvement in your knee function. Notice that it’s not 100%. So, we’re not looking for 100% on a knee replacement, but we’re looking for very good.” E “And I say, ‘Look, these models are based on 12 months because people take 12 months to really get a final result.” A
		Shared decisions	“They don’t want to undergo surgery. And I go, ‘I totally recognize that. Let’s see if there’s any other modalities that we can do.’” D
		Support treatment plan	“I’ll kind of use this [report] as one more piece of information, to kind of support the decision that now is not the time [for surgery], or they’re not a good candidate for a joint replacement.” C
		Report as source of data	“I start talking to the patient based off what I’m seeing [in the report].” G
		Coach behaviour change	“If these are the factors that we can modify, then we need to modify it to get a better outcome.” H “[patients] may not say that they are smokers, or they have emotional health issues, or narcotics use, so sometimes as they keep answering questions, it comes out.” H
		Re-frame progress after surgery	“You were at a 19, and this is where your pain is now. You’re now at a 97. That shows an incredible improvement.” I
	Report content and configuration	Useful content	“…the patients find [pain and function graphs] incredibly helpful because they know how much they hurt, they know what they can do or they can’t do, but they don’t know where they are in comparison to other patients. So I think that’s one of the things this [report] does.” I “I have patients who love this. They love the feedback of it because it gives them one, something tangible that leaves the office, which I think is very useful sometimes.” D
		Not useful content	“So, I probably don’t use [likely need for inpatient care after surgery] very much.” B
	Training needs		“I think helping the surgeons understand how this [report] can help them in addition to how it can help the patients is really what probably most new surgeons need to understand.” I
	Challenges	PROM score interpretation	“I don’t know exactly what that [PCS] means for some patients…[They are] coming in for knee pain, and the knee pain is not too bad but the overall physical function is really bad. Or the opposite. And then, what exactly does that mean?” F
		Workflow in clinic	“We need an army of people of volunteers to get this information from patients.” H “The next visit when they come back, I don’t have [the report] with me and we finished talking about it, so there’s no way for me to kind of check back on it and see if they’ve addressed these things or not.” H “There are some patients who refuse to fill out the tablet if they’re there, or some who never filled it out at home…Some people say no to that.” D “It’s going to be a little complicated to try and keep pulling this form out for every patient because on an average, if I see maybe somewhere like 40 to maybe 50 on a bad day, it would be hard to pull this up.” H
		Burden on patients	“They’re normally bombarded by a lot of things, they’re bombarded by PROMs, they’re bombarded by questionnaires and things like that before even coming into the office when I see them.” D “I still had, over the past couple of weeks, two or three patients coming and asking me that, ‘Did you want me to participate in this?’” H
		Limitations for tailoring report content	“I think the problem is that the data is designed for people with hip arthritis. When you capture people that don’t have hip arthritis, the data doesn’t work correctly.” C
		Contralateral joint pain	Nil presented
		Lack of standardization in what PROMs are used in clinical care	Nil presented
		Whether PROMs are sufficiently useful to warrant implementation costs	“There are still leaders in the field that are arguing that it’s a complete waste of time.” I
	Recommended improvements		“If we could put the A1C on here. Because it’s not just whether or not they have diabetes, but how well controlled it is.” I
Whitebird (2022)	Logistical and perceptual barriers	Logistical barriers	“if it’s five clicks deep nobody’s ever looking at it” (S06) “it’s onerous to access it during the clinic visit” (S10)
		Perceptual barriers	“Patients have no idea what to compare their numbers to” (S07) “I think it would be more confusing for them and it would generate a lot of questions” (S09) “I take all this with a grain of salt because there’s so many overlapping things that could influence the scores” (S07) “there’s just too many confounding variables involved to really tell patients what it really means” (S08)
	Patient-identified outcomes are seen as more valuable than PROMs scores		“That’s probably more meaningful than any of the score data. Are you sleeping through the night? Are you playing with your grandkids? Are you going on a hike? Those are the questions that I think are more meaningful… more useful for the patients than your Oxford score was 45 or 34, or whatever it was” (S03)
	Changes in approaches to using PROMs in clinical care could enhance use and reduce barriers	Patients need to be engaged earlier	“Include it so we can have this information before the patients sign-up for surgery” (S02)
		PROMs could assist in patient communication and decision-making	“could be useful in the preoperative setting…as a tool to help guide the decision to consider surgery more strongly” (S02) “I think that would be good from an expectation standpoint to counsel patients in terms of appropriate expectations after the joint replacement” (S03)
		PROMs could be useful for continuity of care	“If someone else is caring for my patients…having something there for one of the nurses who gets a call about the patients or one of my partners” (S11) “there would be some consistency with that… to incorporate that in whatever type of interaction they’re having with the patient” (OL01).
		Changes to the display of PROMs	“Put it in the medical record in a way that is visually easy to use, and that you could import into your own (patient) note easily” (S06) “pushed to our in-basket when it came through in real time” (S02) “it should be super, super easy and super, super fast” (S06)
	PROMs are widely perceived as valuable in aggregate use	Valuable for organisations or departments	“for the organization, it certainly speaks to the level of care that you deliver…and that patients have great outcomes” (OL05) “it can be used to determine how well the organization is caring [for] and improving these patient-reported outcomes for patients with hip and knee arthritis” (S02) “in a competitive market… having access to that and being able to display our outcomes and patient satisfaction is probably very important to the payers” (S11) “how are we doing as a group…can we do things better?” (S04)
		Valuable for individual surgeons	“I think it, used in aggregate, can be useful for a surgeon in terms of knowing how their patients are doing in general” (S02) “if you don’t measure something, you have no idea how well you’re really doing” (S07) “you want to be able to compare the success of your patients to that of others, which is why you need more of a larger perspective” (S10) “if you’re switching implants or … to compare yourself to some sort of mean and see how you’re performing” (S03)

**Table 3:** Summary of barrier and enabler themes mapped to opportunities and potential actions

Identified barrier themes	Identified enabler themes
B1. Difficulty understanding or interpreting PROMs data	E1. Orthopaedic surgeon focused PROMs education
B2. Orthopaedic surgeon scepticism of the value of PROMs	E2. Tracking postoperative recovery
B3. Logistical issues with using PROMs	E3. Pre- and post-operative patient counselling
B4. Poor accessibility of PROMs data	E4. Guiding clinical decision making
	E5. Orthopaedic surgeon involvement in planning and development of PROMs systems
	E6. Improvements to infrastructure
**Opportunities and potential actions derived from identified barrier and enabler themes**	
Surgeon education (links to B1, B2, E1)	
Interpretation resources at point of care (links to B1, E2, E3, E4)	
Embed information into reporting dashboards (links to B4, E2, E3, E4, E5, E6)	
Dialogue and education around utility and approaches for using PROMs data (links to B2, E1, E2, E3, E4)	
Assessment of technical capabilities and workflow (links to B3, B4, E5, E6)	
Examine processes being used successfully in other settings and/or for other conditions or care (links to B3, B4, E5, E6)	

### Orthopaedic surgeons’ views as derived from the orthopaedic surgeon specific studies

#### Barrier: logistical issues with using PROMs

Logistical issues around using PROMs was a barrier theme identified by two studies specific to orthopaedic surgeons. Whitebird et al. reported that orthopaedic surgeon participants experienced difficulties regarding accessibility and display issues at the point of care: “*Put it in the medical record in a way that is visually easy to use*,* and that you could import into your own (patient) note easily*” [17]. Similarly, Lavallee et al. reported a practical issue with PROMs accessibility due to a high volume patient load: “*It’s going to be a little complicated to try and keep pulling this form out for every patient because on an average*,* if I see maybe somewhere like 40 to maybe 50 on a bad day*,* it would be hard to pull this up*” [18].

#### Barrier: difficulty interpreting/understanding PROMs

A second barrier theme identified by two studies specific to orthopaedic surgeons concerned their limited understanding of interpretation of PROMs scores and how to explain these scores to patients. When considering peer benchmarks, Boyce et al. found that surgeons had difficulty understanding PROMs as identified by their ‘conceptual’ theme (subjective measurement, PROMs versus satisfaction and PROMs versus clinical data) [16]. This theme was similarly reported by Whitebird et al. as ‘perceptual barriers’ to the use of PROMs, with surgeons describing difficulty in helping patients understand PROMs and also surgeon concerns about the impact of potential confounders on PROMs measurement: “*I take all this with a grain of salt because there’s so many overlapping things that could influence the scores*” and “*I think it would be more confusing for them [patients] and it would generate a lot of questions*” [17].

#### Barrier: orthopaedic surgeon scepticism of PROMs

A third barrier theme related to orthopaedic surgeon scepticism or doubts about the value of using PROMs in clinical practice. Lavallee et al. reported on surgeons’ concerns about whether PROMs are warranted given they are sparingly used: “*There are still leaders in the field that are arguing that it is a complete waste of time*” [18]. Similarly, Boyce et al. described orthopaedic surgeon perceptions that the PROMs report they received was not clinically useful and did not impact their practice; concerns were also expressed around cost ineffectiveness and trust in the scientific validity of the PROMs data [16]. This was echoed by Whitebird et al., who reported that surgeons perceived limited utility for PROMs in patient care. They noted that surgeons preferred talking with their patients and using personalised outcomes rather than the use of validated PROMs, perceiving these as difficult to explain [17].

#### Enablers: improvements to infrastructure, surgeon education, aggregate reporting of PROMs data and involving surgeons in system development processes

The three studies specific to orthopaedic surgeons identified several enablers. Boyce et al. reported that enhancements to infrastructure for the collection and dissemination of PROMs data would be beneficial to surgeons. Lavallee et al. referred to the need for surgeon education to aid understanding of PROMs: “*I think helping the surgeons understand how this [report] can help them in addition to how it can help the patients is really what probably most new surgeons need to understand* [18].” Whitebird et al. identified that reporting PROMs scores in their aggregate form may improve surgeon use of PROMs in patient care, and that surgeons should be involved in the planning and development of PROM systems (via end-user testing) to ensure effectiveness, perceived relevance and support clinical use [17].

### Orthopaedic surgeons’ views as derived from the mixed sample studies

#### Barriers: accessibility of PROMs data and difficultly interpreting PROMs data

In the study by Mou et al. [19], barriers to the use of PROMs data by orthopaedic surgeons included obstacles to data access and PROMs implementation: “*it just feels like [the PROMs data] are filling some vault some-place but you never really get access to it*” and “*But I think the hospital… has to be willing to… help to work with the administrative flow or practices because we just can’t do it alone*”. Furthermore, concern about using PROMs as a performance metric was also reported by an orthopaedic surgeon in this study, specifically around inadequate risk adjustment and potential confounders that may be unrelated to surgery. These concepts were consistent with the themes identified in the three studies specific to orthopaedic surgeons.

#### Enablers: guide decision making, postoperative counselling and tracking trajectories

Of the five studies that included mixed health professional samples, one study clearly reported participant quotes that were considered enablers to the use of PROMs by orthopaedic surgeons. Mou et al. reported surgeon’s views around using PROMs to guide clinical decision making: *“The [PROMs] data allow us to see the impact that we’re having with the treatment we offer patients. [They] give patients information about which treatments are most effective* [19].*”* The authors also reported orthopaedic surgeon’s views around the use of PROMs for postoperative counselling: “*It’s extremely useful for the patients to understand where they are on the bell curve… it helps them with expectations as they [recover from surgery]* [19].” Zhang et al. reported that PROMs have the potential to help orthopaedic surgeons track patient trajectories: *“identify patients who don’t follow normative recovery courses or who present later on with new problems”*, however it was noted that following trajectories requires a large volume of patient data [20].

## Discussion

Our systematic review demonstrates there is currently little qualitative evidence on barriers and enablers to orthopaedic surgeon engagement with PROMs data or how orthopaedic surgeons use these data to inform their clinical practice. This paucity of evidence (and the variable quality of evidence) leads to challenges in understanding how to improve the uptake of these data by orthopaedic surgeons. Our focus on identifying the ways in which orthopaedic surgeons engage with PROMs data (and specifically, factors that facilitate or discourage such engagement) is important and timely, given the shift towards measuring outcomes that are important to patients, shared decision-making approaches in orthopaedics and the international adoption of PROMs as indicators for quality improvement.

Quantitative studies involving orthopaedic surgeons provide complementary insights into barriers and enablers but do not provide detailed information. For example, Alshehri et al. [21] conducted a cross sectional survey involving 262 orthopaedic surgeons and determined that while an overwhelming majority were interested in PROMs, a lack of knowledge on how to use PROMs and time restrictions were key barriers to PROMs use in practice. Another cross sectional survey from Souvik et al. [22] involving 87 orthopaedic surgeons reported that 74% of participants perceived an inadequate understanding of how to interpret PROMs data, with half of the sample also acknowledging time constraints as a key barrier. These quantitative study findings also align closely with the main themes identified from our qualitative systematic review, with the previous studies concluding that PROMs-specific education should be further investigated to engage orthopaedic surgeons. Snyder et al. [23] provided further perspective in their multi-institutional survey of orthopaedic surgeons and their care teams. Barriers that impacted PROMs use included difficulties with integrating PROMs into clinical workflow, PROM accessibility challenges, perceptions of poor patient compliance which included concern with patients’ ability to appropriately articulate their outcomes, leading to a mistrust of PROMs data.

Previous systematic reviews have described the use of PROMs by broader groups of health professionals. In 2014, Boyce et al. [14] systematically reviewed studies describing the experiences of multiple types of healthcare professionals in using PROMs information to improve quality of care. They concluded that adequate technology is important for using PROMs as well as specific education on PROMs to assist healthcare professionals with data interpretation. Further, the authors determined that engaging health professionals earlier in the data collection process may facilitate the use of PROMs data. While this 2014 systematic review did not specifically focus on orthopaedic surgeons, and efforts since that time to incorporate education on PROMs data interpretation, as well as strategies for earlier health professional engagement may have progressed since then, the main themes are consistent with those identified in our systematic review. Other published systematic reviews within broader healthcare settings have focused on barriers and enablers to PROMs implementation rather than clinician engagement with or use of PROMs data [24–26].

There were some further insights from other surgical specialities within our systematic review that may also be relevant to orthopaedics. Driscoll et al. [27] included participants from eight surgical specialities and reported that the use of PROMs in clinical practice requires integration of PROMs platforms within existing workflows for surgeon ease. This view was also shared by Mou et al. [19], whose study included participants from five surgical specialities, with the belief that user-friendly systems are needed to facilitate PROMs use by surgeons. An additional finding from other surgical specialities was the desire for key leaders in their respective fields to drive change management processes and ensure successful PROM integration into routine clinical surgical care [27]. Further research is required to determine if a similar approach would facilitate orthopaedic surgeons uptake of and engagement with PROMs data.

It is evident that many practical challenges are impacting orthopaedic surgeon engagement with PROMs data in clinical practice. The studies included in our review were of mixed methodological quality and with risk of bias in most instances. There is a clear need for well-designed studies to further investigate the barriers and facilitators to more fully understand orthopaedic surgeons’ perceptions on the use of PROMs data in clinical practice. Building a more robust evidence base is crucial for informing the development of PROMs reporting systems, practical guidelines and educational resources to improve orthopaedic surgeon engagement with these data.

### Strengths

This systematic review followed established processes in accordance with PRISMA guidelines. We undertook a comprehensive search of the literature (spanning 23 years) across six large databases. Study quality was evaluated using a standardised tool and two reviewers independently conducted each stage of the systematic review process. Our database searches were re-run prior to manuscript preparation to identify any new studies potentially eligible for inclusion. While other reviews have focused on quantitative study designs (which generate comparatively limited insights into constructs such as barriers and enablers), our review is the first to focus on published qualitative research, recognising that this methodological approach can provide unique, in-depth perspectives around orthopaedic surgeons’ views and experiences. We examined studies involving mixed samples of health professionals (where these included orthopaedic surgeons), as well as studies involving only orthopaedic surgeons, to avoid missing potentially relevant data.

### Limitations

We also acknowledge the limitations of this review. Firstly, we only included studies published in English and recognise there may be different perceptions and experiences in countries where English is not the first language. Secondly, we recognise that qualitative studies are not intended to be broadly generalisable (given their relatively small sample sizes, which allow for rich data to be collected) and acknowledge the lack of geographical diversity (most studies were conducted in the USA). Thirdly, only three of the eight included studies focused solely on orthopaedic surgeons. Finally, information on orthopaedic sub-specialities was not available to further characterise the participant samples.

## Conclusion

There is currently a lack of high-calibre qualitative evidence around barriers and enablers to orthopaedic surgeons’ engagement with PROMs data. Additional research is warranted to fully understand factors that influence orthopaedic surgeon engagement with PROMs data and the use of these data in routine orthopaedic care. This is a critical step in developing educational and other supportive resources and guidelines to assist orthopaedic surgeons in using PROMs data within their clinical practice, alongside better integration of these data to improve access at point-of-care.

## Electronic supplementary material

Below is the link to the electronic supplementary material.

**Supplementary Material 1 Table S1**: Search strategies

**Supplementary Material 2 Table S2**: Critical Appraisal Checklist for Qualitative Research - agreed rating from two independent reviewers

## Data Availability

All data are available in this manuscript and supplementary files.

## Abbreviations

PROM: Patient-reported outcome measure
PRISMA: Preferred Reporting Items for Systematic reviews and Meta-Analyses
USA: United States of America
