# Supplementary material for: A systematic review of qualitative studies examining barriers and facilitators to orthopaedic surgeon engagement with patient-reported outcome measures data
Source: J Patient Rep Outcomes. 2024 Dec 18;8:144. doi: 10.1186/s41687-024-00820-x (PMC11655713; doi:10.1186/s41687-024-00820-x)
Supplement: Supplementary file 1 — Supplementary Material 1 Table S1: Search strategies [file 41687_2024_820_MOESM1_ESM.docx]

**Supplementary File Table S1. Search strategies**

|  | **Ovid MEDLINE** | **EMBASE** | **COCHRANE CENTRAL** | **PSYCINFO** | **CINAHL** | **EMCARE** |
| --- | --- | --- | --- | --- | --- | --- |
| 1 | Patient Reported Outcome Measures/ | patient-reported outcome/ | Patient Reported Outcome Measures/ | patient reported outcome measures/ | (MH "Patient-Reported Outcomes") | patient-reported outcome/ |
| 2 | Self Report/ or Health Care Surveys/ | self report/ or health care survey/ | Self Report/ or Health Care Surveys/ | self-report/ | (MH "Self Report") | self report/ or health care survey/ |
| 3 | ((patient* or self) adj (appraisal* or appraised or report* or rated or rating* or based or assess*)).mp. | ((patient* or self) adj (appraisal* or appraised or report* or rated or rating* or based or assess*)).mp. | ((patient* or self) adj (appraisal* or appraised or report* or rated or rating* or based or assess*)).mp. | ((patient* or self) adj (appraisal* or appraised or report* or rated or rating* or based or assess*)).mp. | ((patient* or self) N0 (appraisal* or appraised or report* or rated or rating* or based or assess*)) | ((patient* or self) adj (appraisal* or appraised or report* or rated or rating* or based or assess*)).mp. |
| 4 | (patient* adj (satisfaction or perspective* or experience* or observation* or evaluat*)).mp. | (patient* adj (satisfaction or perspective* or experience* or observation* or evaluat*)).mp. | (patient* adj (satisfaction or perspective* or experience* or observation* or evaluat*)).mp. | (patient* adj (satisfaction or perspective* or experience* or observation* or evaluat*)).mp. | (patient* N0 (satisfaction or perspective* or experience* or observation* or evaluat*)) | (patient* adj (satisfaction or perspective* or experience* or observation* or evaluat*)).mp. |
| 5 | (self* adj (assess* or evaluat* or rating)).mp. | (self* adj (assess* or evaluat* or rating)).mp. | (self* adj (assess* or evaluat* or rating)).mp. | (self* adj (assess* or evaluat* or rating)).mp. | (self* N0 (assess* or evaluat* or rating)) | (self* adj (assess* or evaluat* or rating)).mp. |
| 6 | (client* adj (report* or satisfaction or perspective* or experience* or observation* or evaluat*)).mp. | (client* adj (report* or satisfaction or perspective* or experience* or observation* or evaluat*)).mp. | (client* adj (report* or satisfaction or perspective* or experience* or observation* or evaluat*)).mp. | (client* adj (report* or satisfaction or perspective* or experience* or observation* or evaluat*)).mp. | (client* N0 (report* or satisfaction or perspective* or experience* or observation* or evaluat*)) | (client* adj (report* or satisfaction or perspective* or experience* or observation* or evaluat*)).mp. |
| 7 | (Patient symptom report* or Patient cent*red outcome measure*).mp. | (Patient symptom report* or Patient cent*red outcome measure*).mp. | (Patient symptom report* or Patient cent*red outcome measure*).mp. | (Patient symptom report* or Patient cent*red outcome measure*).mp. | ("Patient symptom report*" or "Patient cent*red outcome measure*") | (Patient symptom report* or Patient cent*red outcome measure*).mp. |
| 8 | (health index* or health indices or health profile*).mp. | (health index* or health indices or health profile*).mp. | (health index* or health indices or health profile*).mp. | (health index* or health indices or health profile*).mp. | ("health index*" or "health indices" or "health profile*") | (health index* or health indices or health profile*).mp. |
| 9 | (measure adj3 recovery).mp. | (measure adj3 recovery).mp. | (measure adj3 recovery).mp. | (measure adj3 recovery).mp. | (measure N2 recovery) | (measure adj3 recovery).mp. |
| 10 | (impact adj2 evaluat*).mp. | (impact adj2 evaluat*).mp. | (impact adj2 evaluat*).mp. | (impact adj2 evaluat*).mp. | (impact N1 evaluat*) | (impact adj2 evaluat*).mp. |
| 11 | (PROMIS or Patient-Reported Outcomes or PROMs).mp. | (PROMIS or Patient-Reported Outcomes or PROMs).mp. | (PROMIS or Patient-Reported Outcomes or PROMs).mp. | (PROMIS or Patient-Reported Outcomes or PROMs).mp. | (PROMIS or "Patient-Reported Outcomes" or PROMs) | (PROMIS or Patient-Reported Outcomes or PROMs).mp. |
| 12 | 1 or 2 or 3 or 4 or 5 or 6 or 7 or 8 or 9 or 10 or 11 | 1 or 2 or 3 or 4 or 5 or 6 or 7 or 8 or 9 or 10 or 11 | 1 or 2 or 3 or 4 or 5 or 6 or 7 or 8 or 9 or 10 or 11 | 1 or 2 or 3 or 4 or 5 or 6 or 7 or 8 or 9 or 10 or 11 | S1 OR S2 OR S3 OR S4 OR S5 OR S6 OR S7 OR S8 OR S9 OR S10 OR S11 | 1 or 2 or 3 or 4 or 5 or 6 or 7 or 8 or 9 or 10 or 11 |
| 13 | Orthopedic Surgeons/ | orthopedic surgeon/ or orthopedic specialist/ | Orthopedic Surgeons/ | Orthopedic Surgeons.mp. | "Orthopedic Surgeons" | orthopedic surgeon/ or orthopedic specialist/ |
| 14 | (orthop?edist* or (orthop?edic adj (surgeon* or doctor* or physician* or professional* or health professional* or healthcare professional* or leader? or practice* or clinic* or department* or unit* or office* or division* or setting*))).mp. | (orthop?edist* or (orthop?edic adj (surgeon* or doctor* or physician* or specialist* or practitioner* or professional* or health professional* or healthcare professional* or leader? or practice* or clinic* or department* or unit* or office* or division* or setting*))).mp. | (orthop?edist* or (orthop?edic adj (surgeon* or doctor* or physician* or professional* or health professional* or healthcare professional* or leader? or practice* or clinic* or department* or unit* or office* or division* or setting*))).mp. | (orthop?edist* or (orthop?edic adj (surgeon* or doctor* or physician* or professional* or health professional* or healthcare professional* or leader? or practice* or clinic* or department* or unit* or office* or division* or setting*))).mp. | (orthop?edist* or (orthop?edic N0 (surgeon* or doctor* or physician* or professional* or "health professional*" or "healthcare professional*" or leader? or practice* or clinic* or department* or unit* or office* or division* or setting*))) | (orthop?edist* or (orthop?edic adj (surgeon* or doctor* or physician* or specialist* or practitioner* or professional* or health professional* or healthcare professional* or leader? or practice* or clinic* or department* or unit* or office* or division* or setting*))).mp. |
| 15 | 13 or 14 | 13 or 14 | 13 or 14 | 13 or 14 | S13 OR S14 | 13 or 14 |
| 16 | 12 and 15 | 12 and 15 | 12 and 15 | 12 and 15 | S12 AND S15 | 12 and 15 |
| 17 | Surgeons/ | surgeon/ | Surgeons/ | surgeons/ | (MH "Surgeons") | surgeon/ |
| 18 | 12 and 17 | 12 and 17 | 12 and 17 | 12 and 17 | S12 AND S17 | 12 and 17 |
| 19 | 16 or 18 | 16 or 18 | 16 or 18 | 16 or 18 | S16 OR S18 | 16 or 18 |
| 20 | "Attitude of Health Personnel"/ | health personnel attitude/ or physician attitude/ | "Attitude of Health Personnel"/ | health personnel attitudes/ | (MH "Attitude of Health Personnel") OR (MH "Physician Attitudes") | health personnel attitude/ or physician attitude/ |
| 21 | ((health or healthcare) adj3 (staff or personnel or professional* or practitioner* or provider*) adj3 (attitude* or belief* or value*)).mp. | ((health or healthcare) adj3 (staff or personnel or professional* or practitioner* or provider*) adj3 (attitude* or belief* or value*)).mp. | ((health or healthcare) adj3 (staff or personnel or professional* or practitioner* or provider*) adj3 (attitude* or belief* or value*)).mp. | ((health or healthcare) adj3 (staff or personnel or professional* or practitioner* or provider*) adj3 (attitude* or belief* or value*)).mp. | ((health or healthcare) N2 (staff or personnel or professional* or practitioner* or provider*) N2 (attitude* or belief* or value*)) | ((health or healthcare) adj3 (staff or personnel or professional* or practitioner* or provider*) adj3 (attitude* or belief* or value*)).mp. |
| 22 | Practice Patterns, Physicians'/ | physician* practice patterns.mp. | Practice Patterns, Physicians'/ | physician* practice patterns.mp. | (MH "Practice Patterns") | physician* practice patterns.mp. |
| 23 | professional practice/ or group practice/ or practice management/ or office management/ or practice management, medical/ or private practice/ | professional practice/ or group practice/ or private practice/ | professional practice/ or group practice/ or practice management/ or office management/ or practice management, medical/ or private practice/ | clinical practice/ or private practice/ | (MH "Professional Practice") OR (MH "Group Practice") OR (MH "Private Practice") OR (MH "Private Practice Management") | professional practice/ or group practice/ or private practice/ |
| 24 | ((professional or clinical or medical or health care or healthcare or surgical or orthop?edic) adj practice).mp. | ((professional or clinical or medical or health care or healthcare or surgical or orthop?edic) adj practice).mp. | ((professional or clinical or medical or health care or healthcare or surgical or orthop?edic) adj practice).mp. | ((professional or clinical or medical or health care or healthcare or surgical or orthop?edic) adj practice).mp. | ((professional or clinical or medical or "health care" or healthcare or surgical or orthop?edic) N0 practice) | ((professional or clinical or medical or health care or healthcare or surgical or orthop?edic) adj practice).mp. |
| 25 | ((surgeon* or orthop?edi*) adj2 (practice* or perceive* value*)).mp. | ((surgeon* or orthop?edi*) adj2 (practice* or perceive* value*)).mp. | ((surgeon* or orthop?edi*) adj2 (practice* or perceive* value*)).mp. | ((surgeon* or orthop?edi*) adj2 (practice* or perceive* value*)).mp. | ((surgeon* or orthop?edi*) N1 (practice* or "perceive* value*")) | ((surgeon* or orthop?edi*) adj2 (practice* or perceive* value*)).mp. |
| 26 | 20 or 22 or 23 or 24 or 25 | 20 or 22 or 23 or 24 or 25 | 20 or 22 or 23 or 24 or 25 | 20 or 21 or 22 or 23 or 24 or 25 | S20 OR S21 OR S22 OR S23 OR S24 OR S25 | 20 or 22 or 23 or 24 or 25 |
| 27 | 19 and 26 | 19 and 26 | 19 and 26 | 19 and 26 | S19 AND S26 | 19 and 26 |
| 28 | ((upper extremit* or upper limb* or shoulder or arm or elbow or hand or lower extremit* or lower limb* or leg or hip or knee or ankle of foot or feet or back or spine or spinal or bone or musculoskeletal or orthop?edic trauma or fracture*) adj (surgeon* or specialist* or physician* or clinician* or professional* or staff or personnel or practitioner* or provider*)).mp. | ((upper extremit* or upper limb* or shoulder or arm or elbow or hand or lower extremit* or lower limb* or leg or hip or knee or ankle of foot or feet or back or spine or spinal or bone or musculoskeletal or orthop?edic trauma or fracture*) adj (surgeon* or specialist* or physician* or clinician* or professional* or staff or personnel or practitioner* or provider*)).mp. | ((upper extremit* or upper limb* or shoulder or arm or elbow or hand or lower extremit* or lower limb* or leg or hip or knee or ankle of foot or feet or back or spine or spinal or bone or musculoskeletal or orthop?edic trauma or fracture*) adj (surgeon* or specialist* or physician* or clinician* or professional* or staff or personnel or practitioner* or provider*)).mp. | ((upper extremit* or upper limb* or shoulder or arm or elbow or hand or lower extremit* or lower limb* or leg or hip or knee or ankle of foot or feet or back or spine or spinal or bone or musculoskeletal or orthop?edic trauma or fracture*) adj (surgeon* or specialist* or physician* or clinician* or professional* or staff or personnel or practitioner* or provider*)).mp. | ((“upper extremit*” or “upper limb*” or shoulder or arm or elbow or hand or “lower extremit*” or “lower limb*” or leg or hip or knee or ankle of foot or feet or back or spine or spinal or bone or musculoskeletal or “orthop?edic trauma” or fracture*) N0 (surgeon* or specialist* or physician* or clinician* or professional* or staff or personnel or practitioner* or provider*)) | ((upper extremit* or upper limb* or shoulder or arm or elbow or hand or lower extremit* or lower limb* or leg or hip or knee or ankle of foot or feet or back or spine or spinal or bone or musculoskeletal or orthop?edic trauma or fracture*) adj (surgeon* or specialist* or physician* or clinician* or professional* or staff or personnel or practitioner* or provider*)).mp. |
| 29 | ((arthroscop* or arthroplast* or joint replacement or joint reconstruction or TJR or TJA) adj3 (surgeon* or specialist* or physician* or clinician* or professional* or staff or personnel or practitioner* or provider*)).mp. | ((arthroscopy* or arthroplast* or joint replacement or joint reconstruction) adj3 (surgeon* or specialist* or physician* or clinician* or professional* or staff or personnel or practitioner* or provider*)).mp. | ((arthroscop* or arthroplast* or joint replacement or joint reconstruction or TJR or TJA) adj3 (surgeon* or specialist* or physician* or clinician* or professional* or staff or personnel or practitioner* or provider*)).mp. | ((arthroscop* or arthroplast* or joint replacement or joint reconstruction or TJR or TJA) adj3 (surgeon* or specialist* or physician* or clinician* or professional* or staff or personnel or practitioner* or provider*)).mp. | ((arthroscop* or arthroplast* or “joint replacement” or “joint reconstruction” or TJR or TJA) N2 (surgeon* or specialist* or physician* or clinician* or professional* or staff or personnel or practitioner* or provider*)) | ((arthroscopy* or arthroplast* or joint replacement or joint reconstruction) adj3 (surgeon* or specialist* or physician* or clinician* or professional* or staff or personnel or practitioner* or provider*)).mp. |
| 30 | 28 or 29 | 28 or 29 | 28 or 29 | 28 or 29 | S28 OR S29 | 28 or 29 |
| 31 | 12 and 26 and 30 | 12 and 26 and 30 | 12 and 26 and 30 | 12 and 26 and 30 | S12 AND S26 AND S30 | 12 and 26 and 30 |
| 32 | ((orthop?edist* or orthop?edic* or joint reconstruction surgeon* or arthroplast* surgeon* or arthroscop* surgeon* or joint replacement surgeon* or musculoskeletal surgeon* or TJR surgeon* or TJA surgeon*) adj9 ("use" or "us?age" or "used" or "using" or utili?e* or utili?ation or utili?ing or need* or felt or incorporat* or experience* or perceiv* or perception* or perspective* or value* or valuable or implement* or barrier* or integrat* or awareness or useful* or familiarity or uptake or benefit* or drawback* or limitation* or opportunit* or challenge* or adopt* or time constraint* or "lack of time" or "time consum*" or time factor* or willing* or daily practice* or daily clinical or clinical practice* or routine* or goals or facilitat* or endors* or tailor* or attitude* or behavio?r* or "point of view" or knowledge or skills or training or engag* obstacle* or enabl* or acceptabilit* or workflow* or work flow* or champion*) adj11 (patient-reported outcome* or PROM? or PROMIS)).mp. | ((orthop?edist* or orthop?edic* or joint reconstruction surgeon* or arthroplast* surgeon* or arthroscop* surgeon* or joint replacement surgeon* or musculoskeletal surgeon* or TJR surgeon* or TJA surgeon*) adj9 ("use" or "us?age" or "used" or "using" or utili?e* or utili?ation or utili?ing or need* or felt or incorporat* or experience* or perceiv* or perception* or perspective* or value* or valuable or implement* or barrier* or integrat* or awareness or useful* or familiarity or uptake or benefit* or drawback* or limitation* or opportunit* or challenge* or adopt* or time constraint* or "lack of time" or "time consum*" or time factor* or willing* or daily practice* or daily clinical or clinical practice* or routine* or goals or facilitat* or endors* or tailor* or attitude* or behavio?r* or "point of view" or knowledge or skills or training or engag* obstacle* or enabl* or acceptabilit* or workflow* or work flow* or champion*) adj11 (patient-reported outcome* or PROM? or PROMIS)).mp. | ((orthop?edist* or orthop?edic* or joint reconstruction surgeon* or arthroplast* surgeon* or arthroscop* surgeon* or joint replacement surgeon* or musculoskeletal surgeon* or TJR surgeon* or TJA surgeon*) adj9 ("use" or "us?age" or "used" or "using" or utili?e* or utili?ation or utili?ing or need* or felt or incorporat* or experience* or perceiv* or perception* or perspective* or value* or valuable or implement* or barrier* or integrat* or awareness or useful* or familiarity or uptake or benefit* or drawback* or limitation* or opportunit* or challenge* or adopt* or time constraint* or "lack of time" or "time consum*" or time factor* or willing* or daily practice* or daily clinical or clinical practice* or routine* or goals or facilitat* or endors* or tailor* or attitude* or behavio?r* or "point of view" or knowledge or skills or training or engag* obstacle* or enabl* or acceptabilit* or workflow* or work flow* or champion*) adj11 (patient-reported outcome* or PROM? or PROMIS)).mp. | ((orthop?edist* or orthop?edic* or joint reconstruction surgeon* or arthroplast* surgeon* or arthroscop* surgeon* or joint replacement surgeon* or musculoskeletal surgeon* or TJR surgeon* or TJA surgeon*) adj9 ("use" or "us?age" or "used" or "using" or utili?e* or utili?ation or utili?ing or need* or felt or incorporat* or experience* or perceiv* or perception* or perspective* or value* or valuable or implement* or barrier* or integrat* or awareness or useful* or familiarity or uptake or benefit* or drawback* or limitation* or opportunit* or challenge* or adopt* or time constraint* or "lack of time" or "time consum*" or time factor* or willing* or daily practice* or daily clinical or clinical practice* or routine* or goals or facilitat* or endors* or tailor* or attitude* or behavio?r* or "point of view" or knowledge or skills or training or engag* obstacle* or enabl* or acceptabilit* or workflow* or work flow* or champion*) adj11 (patient-reported outcome* or PROM? or PROMIS)).mp. | ((orthop?edist* or orthop?edic* or “joint reconstruction surgeon*” or “arthroplast* surgeon*” or “arthroscop* surgeon*” or “joint replacement surgeon*” or “musculoskeletal surgeon*” or “TJR surgeon*” or “TJA surgeon*”) N8 ("use" or "us?age" or "used" or "using" or utili?e* or utili?ation or utili?ing or need* or felt or incorporat* or experience* or perceiv* or perception* or perspective* or value* or valuable or implement* or barrier* or integrat* or awareness or useful* or familiarity or uptake or benefit* or drawback* or limitation* or opportunit* or challenge* or adopt* or “time constraint*” or "lack of time" or "time consum*" or “time factor*” or willing* or “daily practice*” or “daily clinical” or “clinical practice*” or routine* or goals or facilitat* or endors* or tailor* or attitude* or behavio?r* or "point of view" or knowledge or skills or training or engag* obstacle* or enabl* or acceptabilit* or workflow* or “work flow*” or champion*) N10 (“patient-reported outcome*” or PROM? or PROMIS)) | ((orthop?edist* or orthop?edic* or joint reconstruction surgeon* or arthroplast* surgeon* or arthroscop* surgeon* or joint replacement surgeon* or musculoskeletal surgeon* or TJR surgeon* or TJA surgeon*) adj9 ("use" or "us?age" or "used" or "using" or utili?e* or utili?ation or utili?ing or need* or felt or incorporat* or experience* or perceiv* or perception* or perspective* or value* or valuable or implement* or barrier* or integrat* or awareness or useful* or familiarity or uptake or benefit* or drawback* or limitation* or opportunit* or challenge* or adopt* or time constraint* or "lack of time" or "time consum*" or time factor* or willing* or daily practice* or daily clinical or clinical practice* or routine* or goals or facilitat* or endors* or tailor* or attitude* or behavio?r* or "point of view" or knowledge or skills or training or engag* obstacle* or enabl* or acceptabilit* or workflow* or work flow* or champion*) adj11 (patient-reported outcome* or PROM? or PROMIS)).mp. |
| 33 | ((upper extremit* or upper limb* or shoulder or arm or elbow or hand or lower extremit* or lower limb* or leg or hip or knee or ankle of foot or feet or back or spine or spinal or bone or musculoskeletal or orthop?edic trauma or fracture* or TJR or TJA or joint replace* or joint reconstruct*) adj6 (surgeon* or specialist* or physician* or clinician* or professional* or staff or personnel or practitioner* or provider* or care team*) adj9 (("use" or "us?age" or "used" or "using" or utili?e* or utili?ation or utili?ing or need* or felt or incorporat* or experience* or perceiv* or perception* or perspective* or value* or valuable or implement* or barrier* or integrat* or awareness or useful* or familiarity or uptake or benefit* or drawback* or limitation* or opportunit* or challenge* or adopt* or time constraint* or "lack of time" or "time consum*" or time factor* or willing* or daily practice* or daily clinical or clinical practice* or routine* or goals or facilitat* or endors* or tailor* or attitude* or behavio?r* or "point of view" or knowledge or skills or training or engag* obstacle* or enabl* or acceptabilit* or workflow* or work flow* or champion*) adj11 (patient reported outcome* or PROM? or PROMIS))).mp. | ((upper extremit* or upper limb* or shoulder or arm or elbow or hand or lower extremit* or lower limb* or leg or hip or knee or ankle of foot or feet or back or spine or spinal or bone or musculoskeletal or orthop?edic trauma or fracture* or TJR or TJA or joint replace* or joint reconstruct*) adj6 (surgeon* or specialist* or physician* or clinician* or professional* or staff or personnel or practitioner* or provider* or care team*) adj9 (("use" or "us?age" or "used" or "using" or utili?e* or utili?ation or utili?ing or need* or felt or incorporat* or experience* or perceiv* or perception* or perspective* or value* or valuable or implement* or barrier* or integrat* or awareness or useful* or familiarity or uptake or benefit* or drawback* or limitation* or opportunit* or challenge* or adopt* or time constraint* or "lack of time" or "time consum*" or time factor* or willing* or daily practice* or daily clinical or clinical practice* or routine* or goals or facilitat* or endors* or tailor* or attitude* or behavio?r* or "point of view" or knowledge or skills or training or engag* obstacle* or enabl* or acceptabilit* or workflow* or work flow* or champion*) adj11 (patient reported outcome* or PROM? or PROMIS))).mp. | ((upper extremit* or upper limb* or shoulder or arm or elbow or hand or lower extremit* or lower limb* or leg or hip or knee or ankle of foot or feet or back or spine or spinal or bone or musculoskeletal or orthop?edic trauma or fracture* or TJR or TJA or joint replace* or joint reconstruct*) adj6 (surgeon* or specialist* or physician* or clinician* or professional* or staff or personnel or practitioner* or provider* or care team*) adj9 (("use" or "us?age" or "used" or "using" or utili?e* or utili?ation or utili?ing or need* or felt or incorporat* or experience* or perceiv* or perception* or perspective* or value* or valuable or implement* or barrier* or integrat* or awareness or useful* or familiarity or uptake or benefit* or drawback* or limitation* or opportunit* or challenge* or adopt* or time constraint* or "lack of time" or "time consum*" or time factor* or willing* or daily practice* or daily clinical or clinical practice* or routine* or goals or facilitat* or endors* or tailor* or attitude* or behavio?r* or "point of view" or knowledge or skills or training or engag* obstacle* or enabl* or acceptabilit* or workflow* or work flow* or champion*) adj11 (patient reported outcome* or PROM? or PROMIS))).mp. | ((upper extremit* or upper limb* or shoulder or arm or elbow or hand or lower extremit* or lower limb* or leg or hip or knee or ankle of foot or feet or back or spine or spinal or bone or musculoskeletal or orthop?edic trauma or fracture* or TJR or TJA or joint replace* or joint reconstruct*) adj6 (surgeon* or specialist* or physician* or clinician* or professional* or staff or personnel or practitioner* or provider* or care team*) adj9 (("use" or "us?age" or "used" or "using" or utili?e* or utili?ation or utili?ing or need* or felt or incorporat* or experience* or perceiv* or perception* or perspective* or value* or valuable or implement* or barrier* or integrat* or awareness or useful* or familiarity or uptake or benefit* or drawback* or limitation* or opportunit* or challenge* or adopt* or time constraint* or "lack of time" or "time consum*" or time factor* or willing* or daily practice* or daily clinical or clinical practice* or routine* or goals or facilitat* or endors* or tailor* or attitude* or behavio?r* or "point of view" or knowledge or skills or training or engag* obstacle* or enabl* or acceptabilit* or workflow* or work flow* or champion*) adj11 (patient reported outcome* or PROM? or PROMIS))).mp. | ((“upper extremit*” or “upper limb*” or shoulder or arm or elbow or hand or “lower extremit*” or “lower limb*” or leg or hip or knee or ankle of foot or feet or back or spine or spinal or bone or musculoskeletal or “orthop?edic trauma” or fracture* or TJR or TJA or “joint replace*” or “joint reconstruct*”) N5 (surgeon* or specialist* or physician* or clinician* or professional* or staff or personnel or practitioner* or provider* or care team*) N8 (("use" or "us?age" or "used" or "using" or utili?e* or utili?ation or utili?ing or need* or felt or incorporat* or experience* or perceiv* or perception* or perspective* or value* or valuable or implement* or barrier* or integrat* or awareness or useful* or familiarity or uptake or benefit* or drawback* or limitation* or opportunit* or challenge* or adopt* or “time constraint*” or "lack of time" or "time consum*" or “time factor*” or willing* or “daily practice*” or “daily clinical” or “clinical practice*” or routine* or goals or facilitat* or endors* or tailor* or attitude* or behavio?r* or "point of view" or knowledge or skills or training or engag* obstacle* or enabl* or acceptabilit* or workflow* or “work flow*” or champion*) N10 (“patient reported outcome*” or PROM? or PROMIS))) | ((upper extremit* or upper limb* or shoulder or arm or elbow or hand or lower extremit* or lower limb* or leg or hip or knee or ankle of foot or feet or back or spine or spinal or bone or musculoskeletal or orthop?edic trauma or fracture* or TJR or TJA or joint replace* or joint reconstruct*) adj6 (surgeon* or specialist* or physician* or clinician* or professional* or staff or personnel or practitioner* or provider* or care team*) adj9 (("use" or "us?age" or "used" or "using" or utili?e* or utili?ation or utili?ing or need* or felt or incorporat* or experience* or perceiv* or perception* or perspective* or value* or valuable or implement* or barrier* or integrat* or awareness or useful* or familiarity or uptake or benefit* or drawback* or limitation* or opportunit* or challenge* or adopt* or time constraint* or "lack of time" or "time consum*" or time factor* or willing* or daily practice* or daily clinical or clinical practice* or routine* or goals or facilitat* or endors* or tailor* or attitude* or behavio?r* or "point of view" or knowledge or skills or training or engag* obstacle* or enabl* or acceptabilit* or workflow* or work flow* or champion*) adj11 (patient reported outcome* or PROM? or PROMIS))).mp. |
| 34 | 27 or 31 or 32 or 33 | 27 or 31 or 32 or 33 | 27 or 31 or 32 or 33 | 27 or 31 or 32 or 33 | S27 OR S31 OR S32 OR S33 | 27 or 31 or 32 or 33 |
| 35 | limit 34 to english language | limit 34 to english language | limit 34 to english language | limit 34 to english language | S27 OR S31 OR S32 OR S33  Limiters - Published Date: 20000101-20231231; English Language | limit 34 to english language |
| 36 | limit 35 to yr="2000 -Current" | limit 35 to yr="2000 -Current" | limit 35 to yr="2000 -Current" | limit 35 to yr="2000 -Current" | PT Commentary or Doctoral Dissertation or Editorial or Letter or Masters Thesis or Review or Systematic Review | limit 35 to yr="2000 -Current" |
| 37 | limit 36 to "review" | limit 36 to "review" |  | limit 36 to ("comment/reply" or dissertation or editorial or letter or reviews) | S35 NOT S36 | limit 36 to "review" |
| 38 | 36 not 37 | 36 not 37 |  | 36 not 37 |  | 36 not 37 |
| 39 | systematic review.ti,pt. | systematic review.ti,pt. |  |  |  | systematic review.ti,pt. |
| 40 | 38 not 39 | 38 not 39 |  |  |  | 38 not 39 |
| 41 | limit 40 to (case reports or comment or editorial or letter or news or newspaper article) | limit 40 to (conference abstract or editorial or letter) |  |  |  | limit 40 to (conference abstract or editorial or letter) |
| 42 | 40 not 41 | 40 not 41 |  |  |  | 40 not 41 |
