# Supplementary material for: A systematic review of qualitative studies examining barriers and facilitators to orthopaedic surgeon engagement with patient-reported outcome measures data
Source: J Patient Rep Outcomes. 2024 Dec 18;8:144. doi: 10.1186/s41687-024-00820-x (PMC11655713; doi:10.1186/s41687-024-00820-x)
Supplement: Supplementary file 2 — Supplementary Material 2 Table S2: Critical Appraisal Checklist for Qualitative Research - agreed rating from two independent reviewers [file 41687_2024_820_MOESM2_ESM.docx]

**Supplementary File Table S2: Critical Appraisal Checklist for Qualitative Research - agreed rating from two independent reviewers**

|  | **1**  **Philosophical perspective & research methodology** | **2**  **Research methodology & research question/ objectives** | **3**  **Research methodology & methods collect data** | **4**  **Research methodology & representation and analysis data** | **5**  **Research methodology & interpretation results** | **6**  **Statement locating research culturally or theoretically** | **7**  **Influence of the researcher on the research addressed and vice versa** | **8 participants and their voices represented** | **9**  **Ethical / ethics approval** | **10**  **Conclusion flow from the analysis / interpretation of data** |
| --- | --- | --- | --- | --- | --- | --- | --- | --- | --- | --- |
| **Boyce (2014)** | **Y** | **Y** | **Y** | **Y** | **Y** | **Y** | **Y** | **Y** | **Y** | **Y** |
| **Driscoll (2022)** | N | Y | Y | Y | Y | Y | Y | Y | Y | Y |
| **Jansson (2019)** | Y | Y | Y | Y | U | N | N | Y | Y | U |
| **Lavallee (2023)** | N | Y | Y | Y | Y | N | N | Y | Y | Y |
| **Mou (2022)** | Y | Y | Y | Y | Y | N | Y | Y | Y | Y |
| **Rothrock (2019)** | N | N | N | N | N | N | N | N | Y | U |
| **Whitebird (2022)** | N | Y | Y | Y | Y | Y | Y | Y | Y | Y |
| **Zhang (2019)** | N | Y | Y | Y | Y | N | N | Y | Y | U |

Yes = Y

No = N

Unclear = U

(Checklist items extracted from Aromataris et al. **[15]** )
